# HSP-4/BiP expression in secretory cells is regulated by a developmental program and not by the unfolded protein response

**DOI:** 10.1371/journal.pbio.3000196

**Published:** 2019-03-25

**Authors:** Ji Zha, Mingjie Ying, Jasmine Alexander-Floyd, Tali Gidalevitz

**Affiliations:** Biology Department, Drexel University, Philadelphia, Pennsylvania, United States of America; University of Cambridge, UNITED KINGDOM

## Abstract

Differentiation of secretory cells leads to sharp increases in protein synthesis, challenging endoplasmic reticulum (ER) proteostasis. Anticipatory activation of the unfolded protein response (UPR) prepares cells for the onset of secretory function by expanding the ER size and folding capacity. How cells ensure that the repertoire of induced chaperones matches their postdifferentiation folding needs is not well understood. We find that during differentiation of stem-like seam cells, a typical UPR target, the *Caenorhabditis elegans* immunoglobulin heavy chain-binding protein (BiP) homologue Heat-Shock Protein 4 (HSP-4), is selectively induced in alae-secreting daughter cells but is repressed in hypodermal daughter cells. Surprisingly, this lineage-dependent induction bypasses the requirement for UPR signaling. Instead, its induction in alae-secreting cells is controlled by a specific developmental program, while its repression in the hypodermal-fated cells requires a transcriptional regulator B-Lymphocyte–Induced Maturation Protein 1 (BLMP-1/BLIMP1), involved in differentiation of mammalian secretory cells. The HSP-4 induction is anticipatory and is required for the integrity of secreted alae. Thus, differentiation programs can directly control a broad-specificity chaperone that is normally stress dependent to ensure the integrity of secreted proteins.

## Abstract 2

A study in the nematode Caenorhabditis elegans shows that dedicated developmental programs can bypass the requirements for the unfolded protein response during the differentiation of secretory cells, anticipating their future high folding needs.

## Author summary

During differentiation, cells that specialize in secretion of proteins, such as antibody-secreting B cells, prepare for the onset of secretory function by expanding the size of the major secretory organelle, the endoplasmic reticulum (ER), and by increasing the expression of molecular chaperones and folding enzymes. This pre-emptive expansion of the ER depends on activation of the ER stress response pathways and is required for the secretory phenotype. In addition, cells may also need to up-regulate a selected subset of chaperones because different secreted proteins may require different chaperones for their folding and secretion. Except in specialized cases, how this selective up-regulation is achieved, and whether it depends on the ER stress pathways, is not well understood. Using *Caenorhabditis elegans*, we find that a chaperone BiP/HSP-4, which is usually induced in most cells by stress, is selectively induced during differentiation of stem cells into the alae-secreting cells while being repressed in their sister lineage, the hypodermal cells. We find that induction of this chaperone is independent of the known ER stress pathways, while its repression requires a known regulator of development in mammals, BLIMP1/BLMP-1. The pre-emptive induction of BiP/HSP-4 is important for the integrity of secreted alae and cuticle, suggesting that a general molecular chaperone that is a canonical target of ER stress pathways can be selectively regulated by development to ensure the quality of secreted proteome and functionality of the cells postdifferentiation.

## Introduction

Cellular identity is largely defined by the proteins expressed in the cell or cellular proteome, whose functionality depends on successful folding, localization, and functional maintenance of expressed proteins. During cellular differentiation, rapid onset of new protein synthesis challenges the proteostasis and may result in the production of dysfunctional proteins and folding stress if not matched by corresponding increases in required chaperones [1]. This is especially evident for differentiating professional secretory cells because production of large quantities of secreted protein [2,3] makes them extremely sensitive to the folding stress in the endoplasmic reticulum (ER). To accommodate the anticipated increase in newly synthesized proteins, the ER proteostasis networks are expanded during differentiation through activation of the ER stress response [4], known as the unfolded protein response (UPR) [5].

In addition to their expansion, proteostasis networks may need to be remodeled during differentiation since different secreted proteins may require different chaperones for their biogenesis [6,7]. An early example of this was shown during differentiation of a B-cell line into antibody-secreting cells: while expression of the majority of ER proteins, including the Heat Shock Protein 70 (HSP70)-family chaperone BiP, increased in proportion to the expansion of ER size, a small subset of ER proteins was preferentially up-regulated, resulting in their increased local concentration within the ER, presumably to support immunoglobulin folding and secretion [8]. How this selective up-regulation is achieved, and whether it requires the UPR machinery, is not well understood.

The canonical UPR signaling includes three major branches—controlled by the Serine/Threonine-Protein Kinase/Endoribonuclease IRE1 and a basic leucine-zipper transcription factor X-Box Binding Protein 1 (XBP-1), by the Activating Transcription Factor 6 (ATF-6), or by the PRKR-Like Endoplasmic Reticulum Kinase (PERK). IRE1 and/or XBP-1 are essential for differentiation of many secretory cells such as plasma cells [9,10] and eosinophils [11] and for biogenesis of exocrine pancreas and salivary glands in mice [12,13]. IRE1 is an ER transmembrane protein that, upon sensing folding stress in the ER, cleaves the mRNA of XBP-1; the resulting active spliced form of XBP-1 controls expression of molecular chaperones and other ER biogenesis genes [14]. Ectopic expression of spliced XBP-1 in cultured cells is sufficient to induce expansion of the ER size and cell’s secretory capacity, while deletion of *xbp1* gene in the mouse B-cell lineage prevents development of antibody-secreting plasma cells [9,15]. In fact, XBP-1, together with a transcriptional repressor BLIMP1, are the two regulators required for plasma cell differentiation [15,16]. The *xbp1* gene is repressed in resting B cells [17], and BLIMP1 relieves this repression upon B-cell stimulation, leading to up-regulated *xbp1* transcription [15,18]. Thus, plasma cell differentiation program directly regulates the UPR transcription factor responsible for the general increase in the secretory capacity. Indeed, activation of UPR during plasma cell differentiation appears to be in anticipation of increase in secretory load rather than in response to proteostatic stress [19,20].

Compared with the general ER expansion, much less is known about the second aspect of the ER proteostasis remodeling during differentiation: up-regulation of select chaperones to match the cell-type–specific folding needs. Many ER chaperones are expressed in cell- and tissue-specific patterns during development; however, there are only few examples in which the basis of this cell selectivity is understood at the molecular level. One specialized case are the client-specific chaperones, such as a collagen chaperone HSP47, which is normally induced by heat stress but, during development, is co-regulated with its client collagens by developmental transcription factors [21]. An example for the induction of the major stress-responsive ER chaperone BiP can be seen during cardiac development [22]. Unlike the client-specific HSP47, BiP is a broad-specificity chaperone, required for the general housekeeping functions in the ER. The *Grp78* gene (Glucose-Regulated Protein, 78 kDa), which encodes BiP, is a canonical UPR target whose promoter has been used to delineate the UPR signaling and to identify binding motifs for UPR transcription factors [23–25]. The induction of BiP during heart development reflects cooperation between the UPR transcription factor ATF-6 and the cardiac-specific transcription factor GATA-4, which appears to bind *Grp78* promoter through the ER stress element that is otherwise recognized by ATF-6 under stress conditions [22]. It remains unclear whether such cooperation between the UPR and developmental signaling is the rule and how the selective up-regulation of ER chaperones during differentiation is integrated with the cellular differentiation program.

Here, we take advantage of the stereotypical timing and patterns of cell divisions and differentiation in *C*. *elegans* to examine the regulation of a broad-specificity chaperone, the BiP homologue HSP-4, during differentiation of dedicated secretory cells that secrete cuticular ridges called alae. *C*. *elegans* possesses two homologues of BiP: HSP-3, which is both constitutively expressed and stress-responsive, and HSP-4, which has very low basal expression in most cells but is strongly induced by UPR signaling [26,27]. Using the well-characterized transcriptional reporter expressing green fluorescent protein (GFP) from the *hsp-4* promoter (p*hsp-4*::GFP) [28], we find that *hsp-4* is selectively and transiently induced during differentiation of the stem-like seam cells into alae-secreting cells. Asymmetric divisions of seam cells produce anterior daughters that differentiate into hypodermal cells and posterior daughters that continue stem-like divisions but differentiate into the alae-secreting cells after the last division. *hsp-4* is induced only in these posterior cells prior to their differentiation, in an anticipatory fashion. Unexpectedly, this *hsp-4* induction is neither dependent on the three canonical UPR signaling pathways—IRE1/XBP-1, ATF-6, and PERK—nor does it require the known ER stress elements in its promoter. On the other hand, repression of *hsp-4* in the hypodermal-fated cells requires BLMP-1, a *C*. *elegans* homologue of the B-cell differentiation factor BLIMP1. The non-UPR induction of HSP-4/BiP may be selectively required for the folding or secretion of a specific client(s) in alae-secreting cells, as indicated by the abnormal alae structures and compromised barrier function of the cuticle when HSP-4/BiP induction is abolished. Our results demonstrate that a broad-specificity molecular chaperone that is a canonical UPR target can be selectively regulated by developmental signaling, independent of UPR pathways, to ensure the integrity of the secreted proteome and functionality of the cell postdifferentiation.

## Results

### *hsp-4* expression is activated in seam cells prior to their differentiation into alae-secreting cells

Although basal expression of the UPR-inducible BiP homologue HSP-4 is low in most tissues of *C*. *elegans*, the p*hsp-4*::GFP transcriptional reporter is visibly induced in unstressed animals in two highly secretory tissues—spermathecae and the lateral seam. Because seam cells undergo stereotypical and well-characterized divisions and differentiate at defined developmental stages [29], we used them to examine the regulation of BiP expression. During reproductive development, seam cells of V1–V4 and V6 lineages (S1A Fig) undergo two types of divisions—one symmetric division early in the second larval (L2) stage and four rounds of asymmetric divisions [29]. The asymmetric divisions produce anterior daughters that differentiate and fuse with hypodermal syncytium after each cycle of divisions [30] and posterior daughters that continue dividing until the fourth larval (L4) stage, when they differentiate, fuse with each other, and begin secreting proteins to make specialized cuticular structures, named alae [31,32]. In addition to this normal developmental sequence, early L2 animals under certain environmental stress conditions can enter into an alternative developmental program known as dauer diapause, resulting in formation of nonfeeding and long-lived dauer larvae [33]. During dauer development, the seam cells differentiate at the end of the predauer L2 stage, known as L2d stage (S1A Fig), and secrete the dauer-specific cuticle and alae [34].

**Fig 1:**
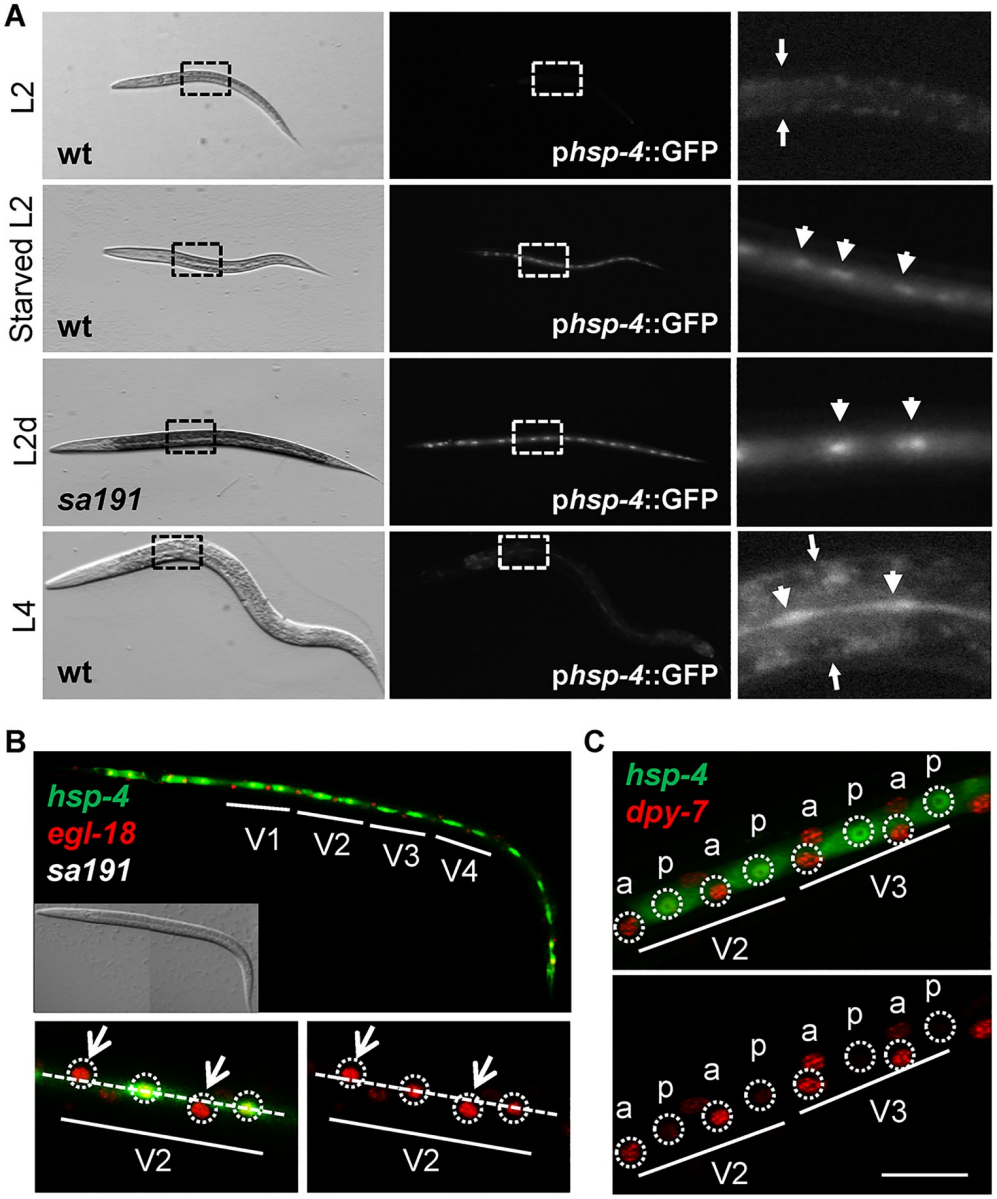
The UPR-inducible BiP homologue HSP-4 is induced during terminal differentiation of seam cells into alae-secreting cells. (A) Transmitted light and fluorescence micrographs of *C*. *elegans* expressing p*hsp*-4::GFP at indicated developmental stages. L2d stage animals are *daf-28(sa191)*. Images are taken at 120× magnification on stereo microscope, under the same imaging conditions. Right panels show enlarged and (for L2 and L4 stages) overexposed boxed areas. Small arrows point to intestine boundaries, arrowheads point to individual seam cells. (B) Confocal image of *daf-28(sa191)* L2d animal expressing p*hsp-4*::GFP and p*egl-18*::H1-mCherry transgenes. Upper panel: projection of z-stack taken through the lateral hypodermis, lower panels: single-plane images. Lower panels show close view of V2 lineage cells, either in both channels or in red channel only, with nuclei circled. Open arrows point to anterior daughter cells, migrating away from the seam center line (dashed line). (C) Close view of V2 and V3 lineage cells of a starved L2 stage WT animal expressing p*hsp-4*::GFP and p*dpy-7*::HIS-24::mCherry transgenes. The anterior (a) and posterior (p) daughters of V2 and V3 seam-cell lineages are indicated, based on *dpy-7* expression. Z-stack projection, scale bar: 20 μm. BiP, immunoglobulin heavy chain-binding protein; *daf-28(sa191)*, an allele causing ectopic L2d entry; GFP, green fluorescent protein; HIS-24, histone; HSP-4, Heat-Shock Protein 4; L2, second larval stage; L2d, predauer L2; L4, fourth larval stage; p*dpy-7*, *dpy-7* promoter, active in hypodermal cells; p*egl-18*, *egl-18* promoter, active in the posterior seam cells after asymmetric divisions; UPR, unfolded protein response; WT, wild type.

We observed that p*hsp-4*::GFP reporter was visibly induced in seam cells during two developmental stages—weakly in the late L4 stage and strongly in L2 stage animals on starved crowded plates (Fig 1A)—while it was undetectable in other larval stages. Since starved L2 animals on crowded plates often initiate the dauer program, we also tested predauer animals. A mutant allele (*sa191*) of an insulin/insulin-like growth factor (IGF)-like protein DAF-28 causes animals to enter the L2d stage even in the presence of food and to remain in that stage for several hours [35,36]. We observed a strong and persistent induction of the p*hsp-4*::GFP reporter in the seam cells of *daf-28(sa191)* animals at the L2d stage (Fig 1A). The reporter induction in the late L4 and predauer animals indicates that seam-specific *hsp-4* expression is triggered at developmental stages that result in differentiation of the alae-secreting cells (S1A Fig).

Closer examination suggested that *hsp-4* expression is indeed induced in the posterior daughter cells, fated to differentiate into alae-secreting cells after the last asymmetric division. To confirm this, we employed two commonly used reporters—p*egl-18*::H1-mCherry, which is preferentially expressed in the posterior cells after asymmetric divisions [37] (S1B Fig), and p*dpy-7*::HIS-24-mCherry, expressed specifically in the anterior cells differentiating into hypodermal cells [38,39] (Fig 1B and 1C). Unexpectedly, the p*egl-18*::H1-mCherry reporter lost its asymmetry in the predauer animals (Fig 1B). However, the p*dpy-7*::HIS-24-mCherry reporter was strongly expressed in *hsp-4*–negative cells and only weakly in *hsp-4*–positive cells in predauers (Fig 1C), confirming that *hsp-4* expression is induced in the posterior seam cells as they are differentiating into the alae-secreting cells.

### *hsp-4* expression is induced in anticipation of differentiation of alae-secreting cells

**Fig 2:**
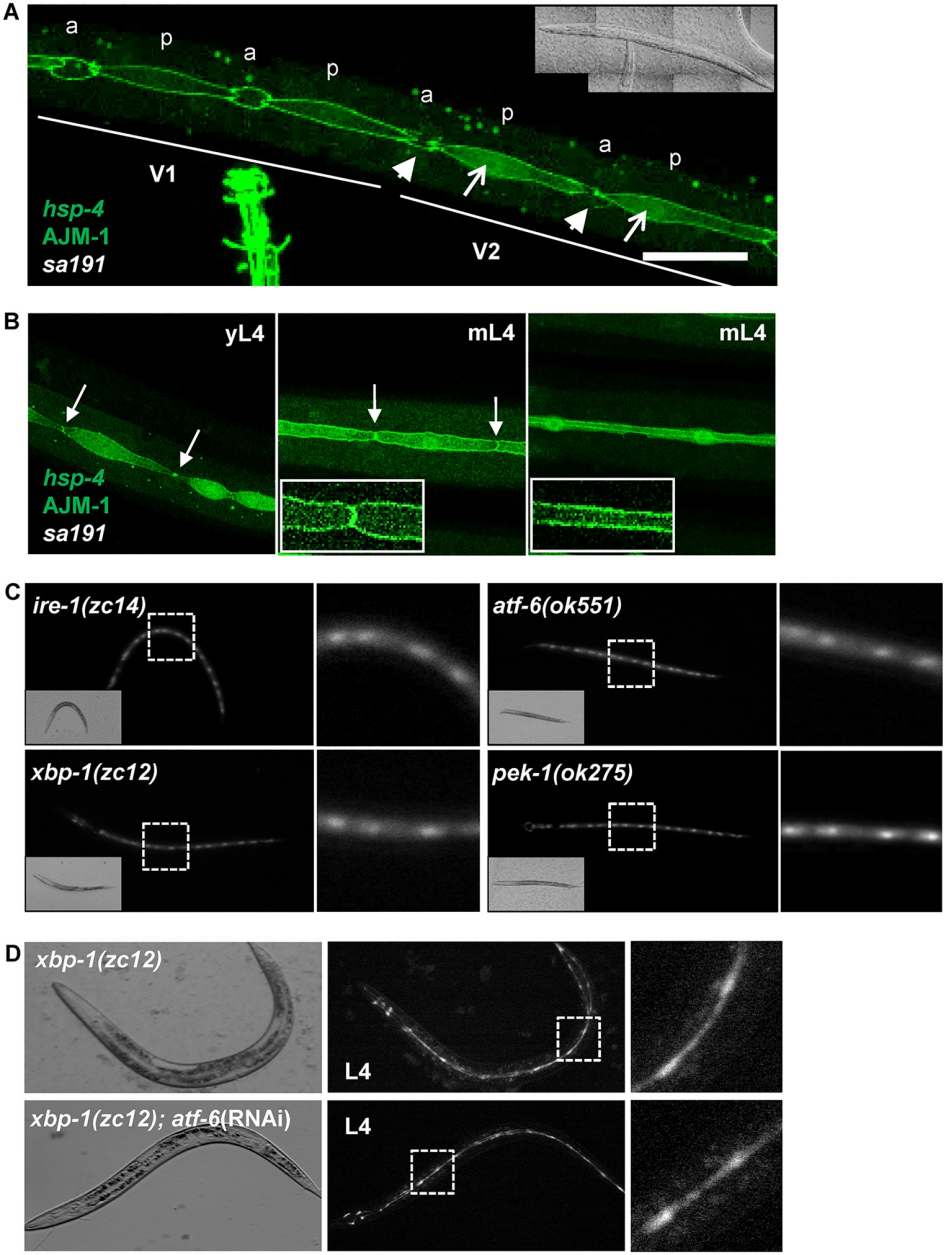
Expression of *hsp-4* is induced early during differentiation, independent from UPR signaling. (A) Confocal images of V1 and V2 lineage cells of a *daf-28(sa191)* early L2d animal (39 hours post-gastrula stage), expressing p*hsp-4*::GFP reporter and AJM-1::GFP fusion protein. Open arrows point to already visible induction of *hsp-4* reporter in posterior daughters (p), which are outlined by the AJM-1::GFP protein; arrowheads point to the remnants of apical junctions of the differentiating anterior daughters (a). Inset: transmitted light, entire animal. Scale bar: 20 μm. (B) Seam cells of WT animals expressing p*hsp*-4::GFP reporter and AJM-1::GFP fusion protein. yL4 stage, left panel; mL4 stage, prior to (middle panel) or after (right panel) seam-cell fusion. Arrows point to the junctions between cells, which disappear after the fusion. Main panels: z-stack projections; insets: single-plane images showing the AJM-1::GFP protein outlining either the cell boundaries prior to fusion (middle panel) or the seam syncytium (right panel). (C) Expression of *hsp-4* reporter in the seam cells of starvation/crowding-induced predauers of indicated UPR-deficient mutant strains. All animals carry the p*hsp-4*::GFP transgene. Imaging as in Fig 1A. (D) Combined loss of XBP-1 and ATF-6 transcription factors does not prevent *hsp-4* induction in differentiating seam cells. Upper panels, *xbp-1(zc12)* animal fed control RNAi (L4440 empty vector); lower panels, *xbp-1(zc12)* animal fed *atf-6* RNAi for two generations. AJM, Apical Junction Molecule; ATF-6, Activating Transcription Factor 6; *daf-28(sa191)*, an allele causing ectopic L2d entry; GFP, green fluorescent protein; HSP-4, Heat-Shock Protein 4; L2, second larval stage; L2d, predauer L2; L4, fourth larval stage; mL4, mature L4 stage animal; RNAi, RNA interference; UPR, unfolded protein response; WT, wild type; XBP-1, X-Box Binding Protein 1; *xbp-1(zc12)*, a UPR-deficient allele of XBP-1; yL4, young L4 stage animal.

While the asymmetric expression pattern showed cell-selective induction of the chaperone BiP/HSP-4 during differentiation, it was not clear whether it was triggered by the postdifferentiation increase in the secretory load or was induced in anticipation of it. To determine how early during the last asymmetric division and differentiation *hsp-4* is induced, we used AJM-1::GFP (Apical Junction Molecule) protein that localizes to apical junctions in epithelial cells and outlines seam-cell boundaries [40]. Immediately after the asymmetric division, AJM-1::GFP is present in both daughter cells, but it is lost from the anterior daughters as they differentiate and fuse with the hypodermal syncytium [41]. In contrast, posterior stem-like daughters continue expressing AJM-1::GFP until they differentiate, when they fuse and begin secreting proteins necessary for the formation of alae [42]. Based on AJM-1::GFP pattern in L2d animals, we determined that induction of *hsp-4* expression in posterior daughters happens already in the early stages after the last division, when anterior daughters have just started to lose their boundaries and have not yet migrated away (Fig 2A). This timing is consistent with anticipatory induction.

To further confirm the anticipatory nature of *hsp-4* induction, we examined its timing in *sa191* animals. Under normal growth conditions at 20 °C, *sa191* animals that do activate the dauer program enter the L2d stage by 41 hours postgastrula. Because the dauer activation is only partial in these L2d animals, most of them (approximately 70%) [36] return to reproductive development several hours later instead of entering dauer [35]. Therefore, most of the *sa191* animals do not complete the seam-cell–differentiation program and do not secrete dauer cuticle or form dauer alae. We found that the seam-cell–specific induction of *hsp-4* expression was readily detectable in 100% (*n* > 100) of *sa191* animals that did enter the L2d stage, assayed at 41 hours postgastrula, and was still present in the same animals at 46 hours postgastrula (see also control animals in *blmp-1* RNA interference [RNAi] experiment below, *n* = 63), after which time many animals return to reproductive development without secreting dauer cuticle or alae proteins.

To ask whether such anticipatory induction early in differentiation is peculiar to the predauer stage, we examined seam cells in L4 animals. The last asymmetric division occurs around the time of the molt from the third larval (L3) stage to the L4 stage; alae-fated cells then differentiate and fuse at the end of the L4 stage, prior to the onset of alae secretion [29,30]. The fusion is detectable by the change in the AJM-1::GFP pattern from outlines of individual seam cells to the outline of the syncytium running along the body length of the nematode (Fig 2B). Because the L4 stage lasts nearly 10 hours at 20 °C, we imaged the seam lineage in young L4 animals after the last asymmetric division and in mature L4 animals prior to and after the fusion. Expression of HSP-4::GFP reporter was evident already in the very young L4 animals (Fig 2B, left panel) well before the fusion event. Collectively, these data show that *hsp-4* expression is selectively and anticipatorily induced during differentiation of the alae-secreting cells.

### Developmental program signals *hsp-4* induction

Since *hsp-4* induction was strongest in predauers and seemed to follow the initiation of the dauer signaling, we asked whether it was responding to a specific dauer-inducing signal. We found that *hsp-4* reporter was similarly induced in predauer animals whether the dauer signaling was induced through the insulin/IGF pathway (*daf-2(1370)* animals) or the transforming Growth Factor β (TGFβ) pathway (*daf-7(e1372)* animals) (S2 Fig). The transcription factor DAF-16 downstream of the insulin/IGF pathway, a *C*. *elegans* homologue of the mammalian FOXO3 (Forkhead Box Protein O3), was recently shown to have an impact on UPR [43], prompting us to ask whether *hsp-4* induction was dependent on DAF-16. Animals bearing a hypomorphic allele *daf-16(mu86)* are dauer deficient; however, those animals that did initiate the predauer program upon starvation/crowding had a p*hsp-4*::GFP induction pattern indistinguishable from the WT (S2 Fig). Finally, dauer induction requires the heat-shock transcription factor HSF-1 [44]. The *hsp-4* gene is heat-inducible, and the *hsp-4* promoter has predicted HSF-1 binding sites (S4A Fig). However, animals carrying the heat-shock-response–deficient *hsf-1(sy441)* allele were still able to induce *hsp-4* in seam cells of starved L2 stage animals (S2 Fig).

Together, these data show that HSP-4/BiP is selectively induced in stem-like seam cells prior to their differentiation into alae-secreting cells. The chaperone induction is anticipatory and is triggered by specific developmental programs—dauer entry or the L4-stage-to-adult transition.

### *hsp-4* induction during seam-cell differentiation is independent from UPR signaling and does not require known ER stress elements in its promoter

Because anticipatory induction of the mammalian homologue of HSP-4 protein, BiP, during differentiation of B cells and other secretory cells is controlled by activation of the UPR signaling, we asked whether other UPR-responsive genes were also activated in the differentiating seam cells. We tested available transcriptional reporters of three genes—*hsp-3*/BiP, *enpl-1*, encoding the orthologue of GRP94 (Glucose Regulated Protein, 94 kDa), and *cnx-1*, encoding the orthologue of calnexin—known to be induced by ER stress in *C*. *elegans* in an IRE-1/XBP-1–dependent manner [45]. Neither *enpl-1* nor *cnx-1* reporters showed detectable induction in the seam cells of either L2d stage (S3A Fig) or late L4 stage animals. The *hsp-3* reporter was constitutively expressed in most tissues and was not induced beyond its basal levels during seam-cell differentiation. Interestingly, expression of *cnx-1* was induced in the V5 seam-lineage–derived neuroblast cells in early L2 animals (S3B Fig). The lack of induction of *hsp-3* or *enpl-1* was not due to the seam lineage being refractory to UPR signaling since we detected induction of both in seam cells when ER stress was induced by treatment with the glycosylation inhibitor tunicamycin (S3C Fig).

We next asked whether UPR pathways were required for selective *hsp-4* induction during seam-cell differentiation. We examined the expression of p*hsp-4*::GFP reporter in starved L2 animals deficient for each of the three canonical UPR pathways by using loss of function alleles (Fig 2C). These alleles were previously characterized as UPR-deficient and were shown to affect the expression of *hsp-4* and other UPR target genes under both ER stress and basal conditions [45,46]. Surprisingly, p*hsp-4*::GFP reporter was induced normally in seam cells despite inactivating mutations of *ire-1*/IRE1 or *xbp-1*/XBP-1 or deletions of *pek-1*/PERK or *atf-6*/ATF-6 (Fig 2C). Mammalian ATF-6 and XBP-1 are both bZIP transcription factors, binding to similar DNA elements and capable of heterodimerization [47]. Genetic inactivation of each is well tolerated in *C*. *elegans*, but loss of both is larval lethal because of the degeneration of the intestine [26]. Thus, it is possible that they compensate for each other in the singly deficient backgrounds. To test this, we used feeding RNAi to down-regulate *atf-6* expression in *xbp-1*–deficient animals. To avoid the possible complications of combining feeding RNAi with starvation, we scored p*hsp-4*::GFP induction during differentiation of seam cells in the L4 stage. All scored (*n* = 20) *xbp-1(zc12)*;*atf-6*(RNAi) animals had normal induction (Fig 2D), despite being unhealthy and with patchy coloration in their intestines, which indicated that RNAi treatment was effective [26].

We could not completely exclude the possibility that a small amount of ATF-6 protein was still expressed in RNAi-treated *xbp-1(zc12)* animals. To address this, we thought to mutate the ER stress elements in the promoter of the *hsp-4* reporter. *hsp-4* promoter was previously found to contain two ER stress element-II–like elements and a putative XBP-1/ATF-6 (cAMP response element [CRE]-like) element [26] (S4A Fig). The *hsp-4* ER stress element-II–like elements ERSE-II, ATTGG-N(6)-CCACA, show some deviation from ERSE-II consensus sequence ATTGG-N(1)-CCAC^G^/_A_, as well as from ERSE consensus CCAAT-N(9)-CCAC^G^/_A_, where CCAAT or ATTGG is a recognition site for the transcription factor Nuclear Transcription Factor Y (NF-Y), while CCAC^G^/_A_ is recognized by XBP-1 or ATF-6 [23,24]. In the *hsp-4* promoter, the two ERSE-II–like elements and their flanking regions contain perfect reverse-complementary sequences such that the region containing these elements, from residue (−584) to (−742), can form a highly stable stem–loop structure (S4A Fig). Because of this unusual arrangement, we chose to delete, rather than mutate, this region. We found that deletion of the ERSE-II-like–containing region did not prevent the induction of *hsp-4* reporter in differentiating seam cells (S4B Fig).

The second ER stress element, between nucleotides (−243) and (−269), is located on the reverse strand (S4A Fig) and contains the TGACGTGT XBP-1/ATF-6 (CRE-like) element, with the core XBP-1 motif underlined. We mutated this element to gGggGTGT (mutated residues in lower case) in the promoter with a deleted ERSE-II–like region, thus eliminating both types of the known ER stress elements in this promoter [26]. In agreement with the lack of effect from deleting UPR transcription factors, elimination of ER stress elements from *hsp-4* promoter did not prevent its induction in posterior daughter cells during seam-cell differentiation (S4B Fig). Surprisingly, this double-mutant promoter was still responsive to induction by ectopically overexpressed spliced XBP-1 (XBP-1s, in neurons [48]). It is possible that additional binding sites, distinct from the known XBP-1 site, exist in this promoter or that XBP-1s activates the mutant promoter through interaction with another transcriptional regulator. However, because of the data from *xbp-1(zc12)*;*atf-6*(RNAi) animals (Fig 2D) and the lack of induction of other UPR target genes (S3A Fig), we favor the conclusion that induction of *hsp-4* expression during differentiation of the seam cells is independent of the three canonical UPR branches.

### BLMP-1, the *C*. *elegans* orthologue of B-lymphocyte–induced maturation protein 1 BLIMP1, represses HSP-4/BiP induction in the hypodermal-fated cells after the terminal division

In addition to the UPR transcription factor XBP-1, the transcriptional regulator BLIMP1 is involved in differentiation of many secretory cell types in mammals, as well as in promoting and maintaining stem cell identity [49]. The *C*. *elegans* orthologue, BLMP-1, is necessary for formation of both adult and dauer alae [50]. Interestingly, the seam-cell divisions themselves are normal in *blmp-1* mutants, suggesting that it only contributes to the postdifferentiation cell fate [51]. Thus, we thought to determine whether BLMP-1 has a role in regulating *hsp-4* induction during seam-cell differentiation. Examination of Model Organism Encyclopedia Of DNA Elements (modENCODE) data [52] showed a strong binding peak for BLMP-1 on the *hsp-4* promoter (S4C Fig). This is likely to represent a true binding peak for two reasons: First, this site does not overlap with the extreme highly occupied target (xHOT) regions, which represent redundant and likely nonspecific binding of multiple transcription factors [53]. Second, we identified a sequence, T**AA****GAAAGC**TCTC**GAAAAGT**C, which is homologous to the known interferon regulatory factor (IRF) elements, near the XBP-1/ATF-6 (CRE-like) element and within the modENCODE peak (S4A Fig; see Materials and methods). Because the mammalian BLMP1 is known to bind with high affinity to the subset of IRF elements containing GAAAG [54], we designate it as a putative BLMP-1–binding site (S4A Fig).

**Fig 3:**
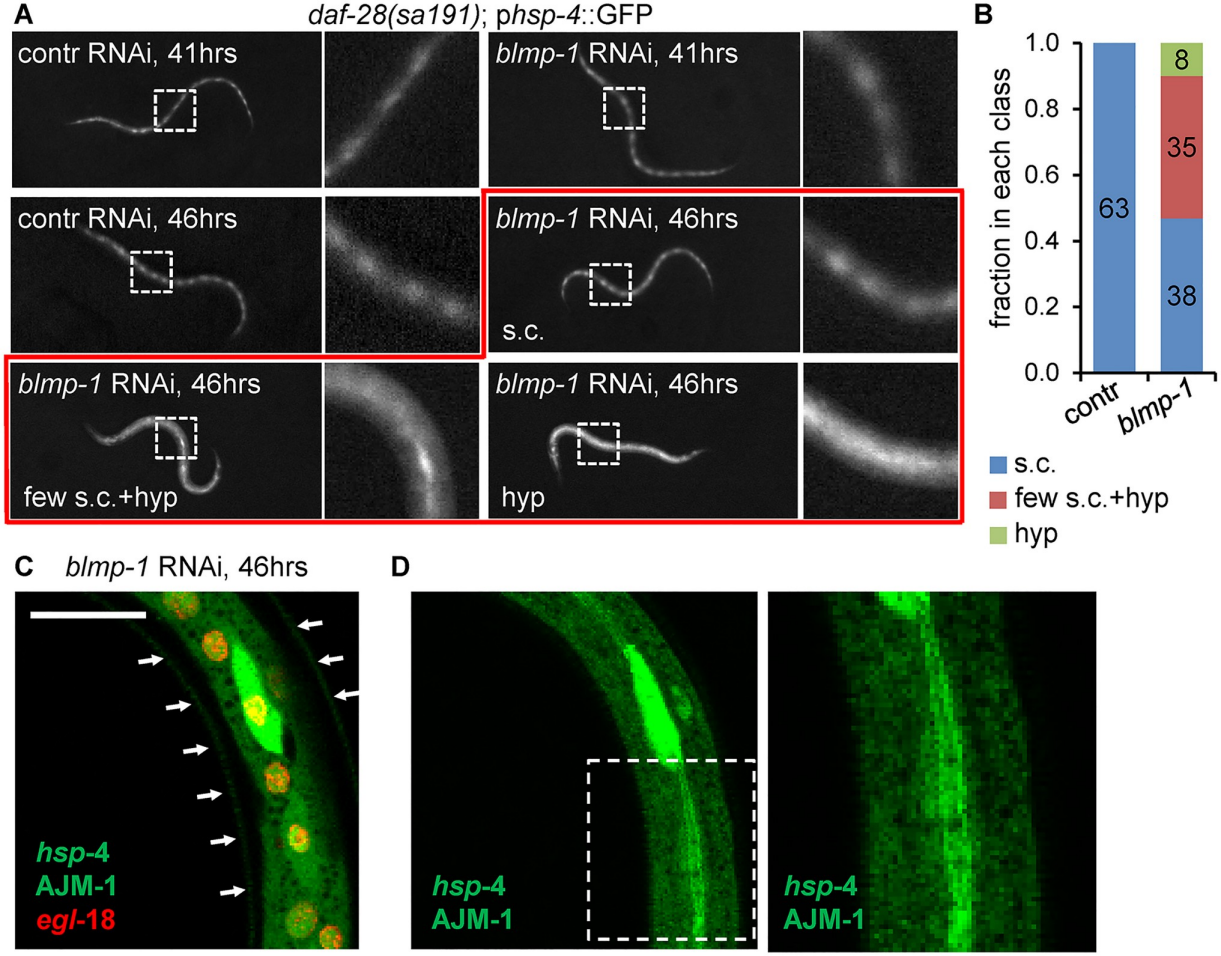
BLMP-1, the *C*. *elegans* orthologue of mammalian transcriptional regulator BLIMP1, represses *hsp-4* induction in the hypodermal lineage during differentiation of the alae-secreting cells. (A) Fluorescence micrographs of *daf-28(sa191)* animals at the early (41 hours postgastrula) and late (46 hours postgastrula) L2d stage. Down-regulation of *blmp-1* expression by feeding RNAi results in strong induction of the *hsp-4* reporter in the lateral hypodermis (indicated in the figure as: hyp) in a fraction of late L2d stage animals, as well as occasional hyperinduction in a few seam cells in addition to the induction in the hypodermis (few s.c. + hyp). An animal with normal induction in seam cells is indicated as (s.c.). Imaging as in Fig 1A. (B) Quantitation of the different *hsp-4* induction classes in animals shown in panel A. Animals that initiated the L2d stage were picked from indicated RNAi plates based on their morphology, using transmitted light only, and then scored for either normal *hsp-4* induction (s.c.), induction in the hypodermis (hyp), or induction in the hypodermis with hyperinduction in some seam cells (few s.c. + hyp). Numbers inside the bars indicate number of animals scored in each class, pooled from 3 independent RNAi experiments. (C, D) Confocal images of a representative animal with (few s.c. + hyp) pattern of *hsp-4* reporter induction following *blmp-1* RNAi. Images show p*hsp-4*::GFP (green), AJM-1::GFP (green), and p*egl-18*::H1-wCherry (red). (C) A single-plane image, taken at a deeper focal plane through the lateral hypodermis, at the level of nuclei. Red signal indicates nuclei of both hypodermal and seam cells. *hsp-4* induction can be seen in the hypodermis; one hyperinduced seam cell is also visible. The arrows outline the animal’s body. (D) Left panel shows a z-projection of the lateral hypodermis in the green channel only, same area as in C. Right panel is a close view of the boxed area, showing a single plane at the level of apical junctions. The AJM-1::GFP outlines the boundary between the seam cell and hypodermis. Scale bar: 10 μm. AJM, Apical Junction Molecule; BLMP-1/BLIMP1, B-Lymphocyte-Induced Maturation Protein 1; contr, control; *daf-28(sa191)*, an allele causing ectopic L2d entry; p*egl-18*, *egl-18* promoter, active in the posterior seam cells after asymmetric divisions; GFP, green fluorescent protein; HSP-4, Heat-Shock Protein 4; L2, second larval stage; L2d, predauer L2; RNAi, RNA interference.

To determine whether the developmental induction of *hsp-4* is dependent on BLMP-1 function, we down-regulated *blmp-1* in *sa191*;p*hsp-4*::GFP animals by RNAi. Under normal growth conditions, *sa191* animals that do initiate the dauer program enter the L2d stage by 41 hours postgastrula. We found no effect of *blmp-1* RNAi on this initial p*hsp-4*::GFP reporter induction at 41 hours postgastrula in all animals that had L2d morphology (Fig 3A, top row). However, by 42 hours, *blmp-1* RNAi caused increased reporter fluorescence in seam cells of these animals, and by 46–47 hours, approximately half of *blmp-1* RNAi animals (*n* = 81) exhibited induction of the reporter in the lateral hypodermis (Fig 3A and 3B). None of the control RNAi animals (*n* = 63) induced *hsp-4* reporter in the hypodermis at any point during the L2d stage. The induction level of p*hsp-4*::GFP reporter in the hypodermis of *blmp-1* RNAi animals was similar to that in seam cells, except for occasional one or few seam cells per animal that exhibited a much brighter further induction (Fig 3).

We considered a possibility that the increase in fluorescence in hypodermal tissue resulted from redistribution of the diffusible GFP protein from posterior seam cells to the hypodermis, if *blmp-1* RNAi caused defects in the seam–hypodermis boundary. However, the GFP fluorescence was contained within the strongly induced cell, and AJM-1::GFP outlines of the seam cells appeared intact (Fig 3C and 3D). Together, these data suggest that the activity that induces the *hsp-4* gene in predauer animals may in fact be triggered in both the hypodermal-fated and alae-fated lineages at this point in development, but the induction of *hsp-4* may at the same time be repressed in the anterior, hypodermal-fated daughter cells by BLMP-1.

We asked whether down-regulation of *blmp-1* would result in induction of other ER chaperone genes. We examined same set of reporters as in S3A Fig, and found that while *hsp-3*, encoding the second BiP homologue, was indeed weakly induced in seam cells of *sa191* L2d animals after *blmp-1* RNAi, the UPR targets *enpl-1*/GRP94 and *cnx-1*/calnexin were unaffected (S5 Fig). Thus, removal of BLMP-1-mediated suppression is not sufficient for the induction of general UPR target genes in the differentiating seam cells, and a BiP-specific inductive factor appears responsible for this developmentally controlled expression of *hsp-4*.

### Loss of HSP-4/BiP expression interferes with structure and barrier function of the cuticle in adults and with alae formation in dauers

**Fig 4:**
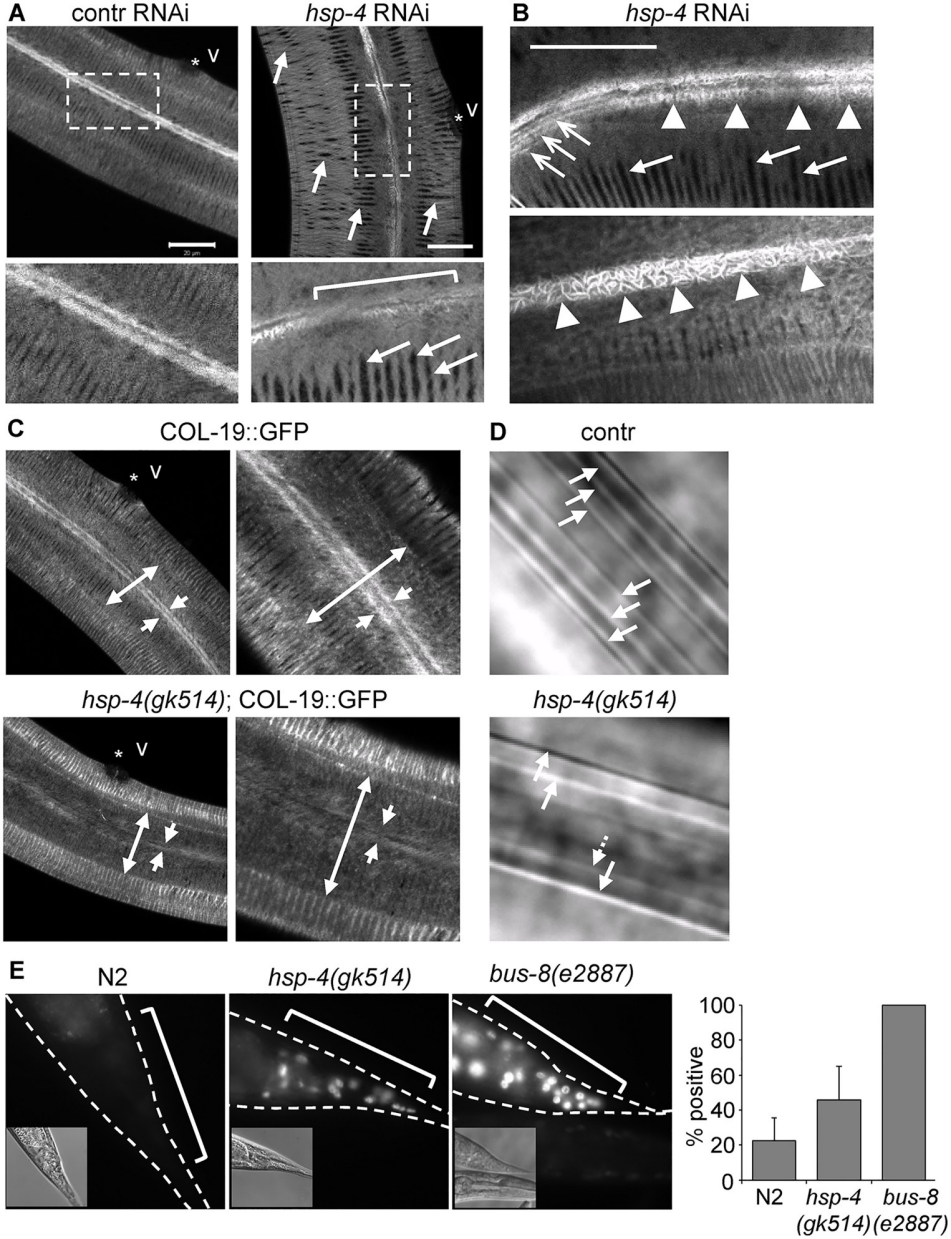
HSP-4/BiP is required for the normal structure and barrier function of the cuticle in adults and alae formation in dauers. (A) Confocal image of COL-19::GFP protein in the hypodermis and alae of a WT (N2) young adult hermaphrodite, fed control RNAi (empty vector, L4440) or *hsp-4* RNAi. COL-19::GFP in controls is deposited in the linear circumferential pattern in the hypodermis, and in linear longitudinal pattern underlying the alae structures. *hsp-4* RNAi results in abnormal deposition of COL-19::GFP protein, with large visible gaps in the circumferential pattern (arrows) and gaps and abnormal appearance of the longitudinal alae pattern (bracket). Star indicates position of the vulva (v). Lower panels: close views of boxed regions pattern. Scale bar: 20 μm. (B) Examples of disrupted COL-19::GFP underlying the alae structures in adult *hsp-4*(RNAi) animals. Open arrows indicate area of normal longitudinal pattern, while arrowheads point to disorganized, “spaghetti-like” pattern of COL-19::GFP. Closed arrows point to the gaps in circumferential pattern. Scale bar: 20 μm. (C) Genetic deletion of *hsp-4* results in decreased deposition of the COL-19::GFP protein in the lateral hypodermis. Double-headed arrows span the lateral hypodermis, overlaying the seam; arrows point to the location of the longitudinal COL-19 structures. (D) Dauer alae are abnormal in *daf-28(sa191)* dauers carrying *hsp-4(gk514)* deletion allele. Arrows indicate individual ridges; the *daf-28(sa191)*;*hsp-4(gk514)* dauer shown here has missing or flattened ridges. DIC images. (E) *hsp-4* deletion increases permeability of the cuticle to small molecules. Staining of nuclei in live animals treated with DNA-binding Hoechst dye reflects the degree of leakiness of the cuticle. Staining was performed as previously described [59]. Percent animals positive for the Hoechst staining is shown as bar graph; data are mean ± SD of three independent experiments. *p* < 0.05 for N2 versus *hsp-4*, *p* < 0.05 for N2 versus *bus-8*, repeated measures ANOVA with Tukey post hoc test. BiP, immunoglobulin heavy chain-binding protein; *bus-8(e2887)*, an allele causing leaky cuticle phenotype; COL-19, *C*. *elegans* cuticular collagen; contr, control; *daf-28(sa191)*, an allele causing ectopic L2d entry; DIC, differential interference contrast; GFP, green fluorescent protein; HSP-4, Heat-Shock Protein 4; RNAi, RNA interference; WT, wild type.

The logic of anticipatory and selective ER chaperone induction during differentiation would suggest that the up-regulated chaperone is required for the specific secretory function of the resulting cell. Yet, BiP is considered to be a broad-specificity rather than client-selective chaperone, consistent with its global induction under folding stress conditions. We asked whether induction of *hsp-4*/BiP expression in differentiating alae-producing cells is important for the postdifferentiation function of these cells by examining the requirements for *hsp-4* for cuticular structure. A GFP-tagged cuticular collagen, COL-19, is expressed starting from the late L4 stage and is normally detected in evenly aligned circumferential pattern, as well as in the longitudinal linear structures of adult alae [55] (Fig 4A–4C). Down-regulation of *hsp-4* by RNAi resulted in a disrupted circumferential pattern in young adults, such that 46% (*n* = 13) of animals contained large gaps between the COL-19::GFP fibers overlaying the lateral hypodermis and those overlaying the ventral/dorsal hypodermis (Fig 4A and 4B). In contrast, only 8% (*n* = 12) of control RNAi animals had gaps in the cuticle (Fig 4A). Furthermore, *hsp-4* RNAi caused occasional areas of disorganization of the longitudinal linear pattern, with COL-19::GFP being deposited in a “spaghetti-like” fashion in some animals (Fig 4B). Similar large gaps and disorganization are known to be caused by mutations in proteins involved in cuticle synthesis and molting [56,57].

Because *hsp-4* and *hsp-3* genes both encode BiP homologues and share a high degree of sequence homology, the *hsp-4* RNAi may target both genes. Thus, we confirmed the HSP-4 requirement for the cuticle using *hsp-4* deletion allele *gk514*. Unstressed *gk514* animals are phenotypically normal and have normal dauer entry rates [36], presumably because of the stress-related role of HSP-4 and because the second BiP homologue, HSP-3, is functionally redundant with HSP-4 [27]. Yet, we found that deletion of *hsp-4* resulted in defects in COL-19::GFP deposition in young adults: the levels of COL-19::GFP over the lateral hypodermis overlaying the seam cells were strongly reduced, and the protein was absent in the longitudinal areas underlying the forming alae (Fig 4C).

Because *hsp-4* induction can be triggered by the dauer developmental program, we examined the alae in dauer cuticles. Because COL-19 is not expressed at these larval stages, we visualized the dauer alae by differential interference contrast (DIC) microscopy. We found that deletion of *hsp-4* resulted in defective formation of dauer alae, with reduced number of ridges and visible gaps in the ridges in all examined dauers (*n* = 5) (Fig 4D).

Finally, we asked whether the structural defects affected the function of the cuticle. Mutations that disrupt cuticle integrity cause it to become permeable to fluorescent dyes, such as Hoechst stain. We found that *hsp-4*(*gk514*) deletion caused increase in the cuticle permeability: although the degree of dye penetration was lower than that in the known leaky-cuticle *bus-8(e2887)* mutant strain [58,59], twice as many *hsp-4*(*gk514*) as WT N2 animals took up the dye (Fig 4E). Thus, HSP-4 protein is required for the formation of the structurally and functionally intact cuticle, and this function of HSP-4 is not compensated for by HSP-3.

## Discussion

The importance of UPR signaling in the general expansion of ER biosynthetic capacity during differentiation of secretory cells has been firmly established. However, it is unclear whether UPR coordinates the repertoire of ER chaperones to the folding needs of specific secretory cell types. We find that during differentiation of the alae-secreting cells, induction of the stress-responsive *C*. *elegans* BiP homologue, HSP-4, bypasses the requirement for the canonical UPR signaling. Instead, HSP-4/BiP is induced by specific developmental programs—the dauer program or L4-stage-to-adult transition. Interestingly, induction of HSP-4/BiP in the hypodermal-fated cells is repressed at the same developmental stage by a known transcriptional regulator of development, BLMP-1/BLIMP1, which also regulates differentiation of many secretory cell types in mammals. Importantly, induction of HSP-4 is not required for the differentiation of alae-secreting cells, per se, but is essential for the secretory function of these cells postdifferentiation.

Sharp increases in BiP expression are often interpreted to indicate activation of the UPR and are thought to require ER stress elements in its promoter. However, under some pathological conditions, the regulatory mechanisms differ from this expectation. For example, increased expression of BiP and other chaperones during acute-phase response in mice with bacterial infection was regulated by binding of Signal Transducer and Activator Of Transcription 3 (STAT3) directly to *Gpr78* promoter [60]. Even under conditions of ER stress, caused by limitation of specific folding resources, induction of the canonical UPR target proteins including BiP can be either dependent or independent of UPR signaling [61]. Induction of *hsp-4* expression in our study may indicate the action of a non-UPR transcription factor(s), specific to the differentiation of alae-secreting cells.

Another possibility is that a member of stress-responsive family of CREB (CRE binding)/ ATF transcription factors, other than ATF-6, is involved in regulating *hsp-4*, similar to the regulation of *Grp78* by ATF-4 during translation block [62] or involvement of another CREB transcription factor, Old Astrocyte Specifically-Induced Substance (OASIS), in bone development [63]. However, mutating the CRE-like element in *hsp-4* promoter did not disrupt the pattern of its induction, making this possibility unlikely. Finally, it is possible that *hsp-4* is regulated during alae-secreting cell differentiation by a UPR transcription factor binding to an element other than the known ER-stress elements. This is an intriguing possibility since we do see induction of the mutant *hsp-4* transcriptional reporter, lacking ER-stress elements, in neurons expressing spliced XBP-1.

The transcriptional induction of *hsp-4* gene during differentiation of alae-secreting cells appears to be anticipatory relative to its putative client protein(s). In L4 stage larvae, *hsp-4* induction precedes the fusion and differentiation by several hours, while in predauers, we first detect the p*hsp-4*::GFP reporter fluorescence soon after the last asymmetric division, before the anterior daughters move away from the seam. Considering the time needed to accumulate the fluorescent signal to detectable levels, activation of *hsp-4* promoter is likely to occur even earlier. Even more strikingly, most *sa191* animals will have returned to reproductive development at 20 °C instead of entering dauer and thus will not have finished differentiation of the alae-secreting cells; yet, all *sa191* L2d animals strongly induce the *hsp-4* reporter. The anticipatory induction here parallels the regulatory logic of the UPR induction during differentiation of secretory cells in mammals [19,20], even though it appears to bypass the UPR. It would be interesting in the future to understand whether this difference is an example of different organisms or even different cell types using different routes to achieve the same goal (timely increase in the necessary chaperone), or whether it reflects the difference between the need for the generic expansion of ER capacity versus the need to match the chaperone repertoire to the cell-specific proteome.

HSP-4 induction is also not simply a consequence of asymmetric divisions because it is not induced after asymmetric seam-cell divisions in other developmental stages. Together with the absence of a generic UPR in these cells and with apparent independence of *hsp-4* induction from the canonical UPR signaling, these data suggest that the early differentiation program that determines the identity of the posterior daughter cell is able to directly regulate this chaperone. This phenomenon is similar to the recently reported regulation of some cytoplasmic chaperones, required for the myofilament formation, by the helix–loop–helix protein HLH-1 (a *C*. *elegans* orthologue of the Myogenic Differentiation transcription factor MyoD) during embryonic muscle differentiation in *C*. *elegans* [64]. Similarly, the transcription factor Kruppel-like zinc finger protein 9 (Zf9) that regulates collagen-specific chaperone HSP47 in fibrotic tissues is capable of binding a collagen promoter [21]. In these examples, the chaperones and their clients are regulated by the same transcription factor(s). While we do not know whether a similar regulatory logic applies to the developmental regulation of the *hsp-4* gene since transcription factors that specify the identity of the alae-secreting cells are unknown, our data do show that HSP-4 function is specifically required for the alae-secreting function of these cells postdifferentiation.

Another aspect of the observed temporal control of *hsp-4* transcription is its repression in the lateral hypodermis by BLMP-1. Silencing of *blmp-1* resulted in *hsp-4* reporter expression in the anterior, hypodermal-fated daughter cells. Interestingly, this ectopic induction of *hsp-4* in *blmp-1*(RNAi) animals was observed only following the last division before alae-secreting cells are specified but not during asymmetric seam-cell divisions in other larval stages. The most facile explanation for such pattern of induction is existence of a positive inductive signal that is activated in the entire seam lineage at the onset of differentiation in L2d or L4 stage animals. In such a case, the combination of this inductive signal in the entire seam lineage with the repressive action of BLMP-1 in the hypodermal-fated cells may explain the selective *hsp-4* induction in the posterior daughter cells. This is also consistent with de-repression of *hsp-4* in the anterior, hypodermal-fated daughters in *blmp-1*(RNAi) animals. Alternatively, the inductive signal may be specific to the posterior daughters as they assume the alae-secreting fate. In this case, *hsp-4* induction in the hypodermis upon *blmp-1* RNAi may reflect de-repression of a different factor that can induce *hsp-4* expression. Because our promoter sequence analysis suggests possible direct binding of BLMP-1 to the *hsp-4* promoter, we favor the former scenario.

The dependence of the cuticle functionality on HSP-4 is somewhat surprising, given that the second BiP homologue, HSP-3, is basally expressed in seam cells. BiP is considered to be a broad-specificity chaperone, capable of binding the majority of proteins that are folded in the ER [65,66], and HSP-4 protein in most *C*. *elegans* cells is only induced under folding stress conditions, further supporting the idea of nonselectivity of its function. Yet, its induction specifically in the alae-secreting cell precursors, and the alae and cuticle defects seen with its deletion suggest a unique cell-specific requirement for this chaperone. One possibility is that certain secreted proteins expressed in these cells require HSP-4, but not HSP-3, for their folding and secretion. Although HSP-4 and HSP-3 proteins are highly conserved and thought to be largely functionally redundant [27], they are not identical, with 83% identity and 97% similarity in their peptide-binding domains. Another, less likely, possibility is that HSP-4 has a unique function in these cells, unrelated to its binding of unfolded proteins.

While the lack of *hsp-4* induction has clear negative consequences for the cuticle secretion, the functional importance of *hsp-4* repression by BLMP-1 in the hypodermal-fated cells is not immediately clear. Deletion of *blmp-1* was previously shown to cause defective formation of alae, and *blmp-1*–deficient animals have oxidative-stress–sensitive cuticles and a dumpy appearance [67,68], indicating global cuticle defects. Because the *blmp-1* deletion is not cell-specific, we do not know whether these defects stem from functional deficiencies in the lateral hypodermis, where *hsp-4* is de-repressed in the absence of BLMP-1. However, it is possible that inappropriate induction of HSP-4 in hypodermal cells results in their decreased ability to secrete proteins because overexpression of a broad-specificity BiP chaperone under nonstress conditions and in the absence of a high-affinity client may nonspecifically stabilize folding intermediates and decrease rates of folding in the ER. Indeed, overexpression of BiP in Chinese hamster ovary (CHO) cells blocks secretion of a subset of proteins, while overexpression of its cytosolic counterpart, HSP70, causes developmental delays in *Drosophila* [69,70]. In addition to individual chaperones, ectopically increased UPR activity can be detrimental to animal development [71], and different tissues may have different tolerance levels [48].

The integration of developmental and stress signaling is emerging as an important contributor to multiple aspects of metazoan biology [5,72,73]. UPR signaling pathways can be specifically activated in the absence of ER stress, for example, by growth factor signaling or infections: IRE1 can be activated by internalized Vascular Endothelial Growth Factor (VEGF) receptor 2 through direct interaction [74], while Toll-like receptors (TLRs) in macrophages activate it by a NADPH oxidase-dependent signal [75]. Interestingly, the TLR-induced IRE1 activation does not result in chaperone expression or ER expansion, as would be expected from stress-activated IRE1, but rather promotes sustained production of inflammatory mediators [75]. Similarly, a canonical UPR transcription factor, ATF-6, and other members of the CREB/ATF family respond to extracellular cues in osteoblasts and odontoblasts by regulating expression of collagens and other matrix-forming proteins [63,76], presumably by interacting with cell-type–specific transcriptional machinery. Thus, physiological processes can not only induce the generic UPR activation but can also trigger specific UPR pathways and, remarkably, control their outcomes. Our data show that, in addition, developmental signals can control the repertoire of induced chaperones directly, bypassing the UPR. Delineating the mechanisms integrating the physiological and stress signaling will thus be instrumental to further our understanding of the regulation of development, the pathogenesis of developmental disorders, and the mechanisms that maintain organismal homeostasis.

## Materials and methods

### Strains and genetics

Standard methods were used for worm culture and genetic crosses [77]. After crosses, strains were confirmed by PCR and restriction digest or sequencing. Animals were synchronized by picking gastrula-stage embryos from well-fed uncrowded plates.

The following strains were obtained from the *Caenorhabditis* Genetics Center (CGC): SJ4005(*zcIs4*[p*hsp-4*::GFP]), SJ30(ire-1(zc14) II; *zcIs4*[p*hsp-4*::GFP]), SJ17(*xbp-1*(*zc12*) III), RB772(*atf-6*(*ok551*) X), RB545(*pek-1*(*ok275*) X), VC1099(*hsp-4(gk514)*II), JT191(*daf-28(sa191)*V), RW11606(*unc-119*(*tm4063*) III; *stIs11606* [*egl-18a*::*H1-wCherry* + *unc-119*(+)]), SD1546(ccIs4251 I; stIs10166 [*dpy-7*p::HIS-24::*mCherry* + *unc-119*(+)]), PS3729(*unc-119*(*ed4*) III; *syIs78*[AJM-1::GFP + *unc-119*(+)]), CB1372(*daf-7*(*e1372*) III), CF1038(*daf-16*(*mu86*) I), CB1370(*daf-2*(*e1370*) III), PS3551(*hsf-1*(*sy441*) I), TP12(*kaIs12*[COL-19::GFP]), and CB6208(*bus-8*(*e2887*) X).

BC10514(*dpy-5*(*e907*) I; *sEx10514* [*rCesT05E11*.*3*::GFP *+ pCeh361*]) and BC10700(*dpy-5*(*e907*) I; *sEx10700* [*rCesZK632*.*6*::GFP + *pCeh361*]) strains and a strain expressing p*hsp-3*::YFP were a gift from the Morimoto lab (Northwestern University, Evanston, IL, USA). WT (N2) animals were a subclone of N2Bristol from the Morimoto Lab.

Primers used for PCR or to sequence-verify crosses with UPR mutant alleles were as follows. For *hsp-4(gk514)*: hsp-4_Ext_F:CCTCCGATTACTCCTGCTTG; hsp-4_Int_F:GTTTGATGCTGGGTTGACAAAG; and hsp-4_Ext_R:GAGTCTTCAAGAATGGGCGAG. For *ire-1(zc14)*: ire-1(zc14)_F:ATCAGCCAACGACCAATCTGC and ire-1(zc14)_R:GAAGCTTTGGATGGGCGAATAG; the mutation was confirmed by digesting the PCR product with BstBI. For *atf-6(ok551)*: atf-6_Ext_F:ATACCGCGTCAAGGAATCAC; atf-6_Int_R:TTAAATCTCACGCAGGCAAG; and atf-6_Ext_R:AATTGGCCAGTCCCTGTCAC. For *pek-1(ok275)*: pek-1_Ext_F:TCGGAGCACACGATTTCTCG; pek-1_Int_R:CTTGTGGACCCGGAGATACG; and pek-1_Ext_R:CTGAGCACATCTGACGTAAG.

### Generation of *sa191* and starved predauer animals

To generate L2d stage animals in *daf-28(sa191)* genetic background, appropriate strains were grown at 20 °C under noncrowded/noncontaminated conditions on fresh plates seeded with OP50 *Escherichia coli* for at least 2 generations. 20–40 YA animals were then picked to fresh plates, and L2d stage larvae were picked among their progeny based on their morphology [33,36]: L2d animals are radially constricted, although to a lesser extent than dauers; they are larger than L2 animals but with the germline morphology of L2 stage; they have a uniformly dark intestine; and they exhibit slow pharyngeal pumping. A similar procedure was used for other developmental stages. To generate L2d animals by starvation/crowding, parents were placed on fresh plates seeded with OP50 *E*. *coli* at 20 °C, and plates were examined daily until there was no food left. Predauers were imaged 1–2 days later.

### Transgene construction

p*hsp-4*::GFP-containing plasmid (#21896) was obtained from Addgene (Watertown, MA, USA). A 54-bp vector-derived region between the end of the *hsp-4* promoter and the start of the GFP, which incidentally contained a PQM-1/DAE-like element, was removed using restriction enzyme PpuMI. To construct the p*hsp-4*-ER stress element-II(del)::GFP transgene, 171 bp of the ER stress element-II–like region was deleted using Q5 Site-Directed Mutagenesis Kit (New England Biolabs, Ipswich, MA, USA). To construct p*hsp-4-ER stress element(del)-xbp-1(mut)*::GFP, the XBP-1/ATF-6 element in the *ER stress element(del)* promoter was mutated from GATGACGTGT to GAgGggGTGT. All constructs were verified by sequencing (Macrogen, Rockville, MD, USA). The mutagenesis primers were as follows: ESERII_del_F:CGGGTCTCTAAGGAAAGGATTC; ESERII_del_R:CCCAGTTGGACATCGGGTC; XBP_1_F:CCTCTCCGATAAGTACACGTTGC; XBP_1_R:GGGTGTATTAGTGCTGGAGAAATC. Transgenes were injected as a mix of 20 ng/μL plasmid DNA and 80 ng/μL sonicated salmon sperm DNA.

### RNAi

The RNAi clones were from the Ahringer library. For RNAi experiments, animals were grown for one or two generations on 0.4 mM-IPTG–containing plates, spotted with designated RNAi bacteria. For *atf-6* RNAi, *xbp-1(zc12)*;*phsp-4*::GFP animals were imaged at the L4 stage. For *hsp-4* RNAi, COL-19::GFP deposition was examined in young adult animals. For *blmp-1* RNAi, 15–20 L4 stage progeny of RNAi-treated *daf-28*(*sa191)*;*phsp-4*::GFP parents were placed on fresh RNAi plates, gastrula-stage embryos were picked 1–2 days later, and L2d stage animals were scored 41–46 hours later. All experiments were repeated with a different population of animals 2–3 times.

Prior to the experiments, the RNAi plates were tested for the expected phenotypes, such as, for example, larval arrest of second generation of *xbp-1* mutant animals on *atf-6* RNAi, to ensure proper induction of the RNAi.

### Identification of a putative BLMP-1-binding site

A sequence T**AA****GAAAGC**TCTC**GAAAAGT**C is located within the modENCODE BLMP-1 peak on the *hsp-4* promoter, near the XBP-1/ATF-6 (CRE-like) element (S4A and S4C Fig). This sequence is homologous to the known IRF binding site. The sequence contains a perfect match to the IRF consensus sequence GAAA^G^/_C_^T^/_C_ found in the MHC class I promoter (underlined) and partial matches (in bold) to an interferon-stimulated response element, found in most interferon-inducible promoters (^A^/_G_NGAAANNGAAACT), and to the positive regulatory domain (PRD) element found in the INF-β promoter (G(A)AAA^G^/_C_^T^/_C_GAAA^G^/_C_^T^/_C_) [78]. Because the mammalian BLMP1 is known to bind with high affinity to the subset of these elements containing GAAAG [54] and the IRF consensus sequence in the *hsp-4* promoter contains this core sequence, we designated it as a putative BLMP-1-binding site (S4A Fig).

### Microscopy

#### Confocal

Animals were mounted on 2% agar pads, immobilized with sodium azide, and imaged with Zeiss LSM700 microscope, using 1.4NA 63x oil objective. Where indicated, 12-bit confocal z-stacks were reconstructed in ImageJ as 3D projections.

#### Stereo

Animals were mounted as above or immobilized by chilling on plates. Imaging was performed with a Leica M205FA microscope (Leica, Wetzlar, Germany) and Hamamatsu Orca R2 camera (Hamamatsu, Hamamatsu City, Japan), keeping magnification and intensity of fluorescence source (Chroma PhotoFluor 2; 89North, Williston, VT, USA) constant within each experiment.

## Supporting information

S1 Data Primary data used to generate the bar graphs in all figures. (XLSX)

S1 Fig Seam-cell divisions in *C*. *elegans*. (A) Schematic diagram of cell divisions in V1–V4 and V6 seam lineages during postembryonic development. Positions and lineages of seam cells in L1 stage larvae are indicated in and above the worm outline. Left diagram corresponds to reproductive development, right to dauer development. After most asymmetric divisions, anterior daughter cells (a) fuse with the hyp7 hypodermal syncytial cell, while posterior (p) daughters retain their stem-like seam fate. After the last asymmetric division in the L4 stage, posterior daughters initiate terminal differentiation into the alae-secreting cell, fuse with each other, and begin to secrete alae-constituents and other cuticular proteins (indicated by blue horizontal lines). Additionally, posterior daughters may undergo differentiation into alae-secreting cells at the end of the L2d stage, resulting in secretion of the dauer cuticle (green horizontal lines); these cells do not fuse and resume asymmetric divisions in PD animals. (B) p*egl-18*::mCherry transgene in L1–L2 stage *daf-28(sa191)* animals. Left panel, L1 animal (20 hours postgastrula) shows differential expression of *egl-18* reporter in seam cells following the first asymmetric division. Anterior (a) and posterior (p) daughters are indicated. Right panel, early L2 animal (26 hours postgastrula) following the symmetric division, with similar *egl-18* expression in anterior and posterior daughters; stars indicate anterior daughters from the previous round of division in the L1 stage. *daf-28(sa191)*, an allele causing ectopic L2d entry; p*egl-18*, *egl-18* promoter, active in the posterior seam cells after asymmetric divisions; L1, first larval stage; L2, second larval stage; L2d, predauer L2; L4, fourth larval stage; PD, postdauer.
(TIF)

S2 Fig *hsp-4* induction during seam-cell differentiation in predauers responds to the general dauer entry program. Fluorescence micrographs of predauer animals of indicated mutant strains, expressing p*hsp-4*::GFP. *daf-7(e1372)* mutant animals enter the L2d stage at 20 °C, similar to the *daf-28(sa191)* animals; in other strains, the predauer stage was induced by starvation/crowding. Imaging as in Fig 1A; right panels are close views of the boxed areas. *daf-28(sa191)*, an allele causing ectopic L2d entry; GFP, green fluorescent protein; HSP-4, Heat-Shock Protein 4; L2, second larval stage; L2d, predauer L2.
(TIF)

S3 Fig *hsp-4* induction during seam-cell differentiation does not reflect a generic UPR induction. (A) Confocal micrographs of *daf-28(sa191)* L2d animals carrying indicated transgenes. Upper panels are projections of confocal stacks through half of the animal, overlaid on a transmitted light image; middle and bottom panels show projections of confocal stacks through the middle of the body or through the hypodermal layer. AJM-1::GFP protein marks apical junctions and outlines seam-cell boundaries (small closed arrows in the bottom panels). Open arrows point to various cells showing induction of the transcriptional reporters for indicated UPR-target genes (*hsp-3*, *enpl-1*, and *cnx-1*, coding for *C*. *elegans* orthologues of BiP, GRP94, and calnexin, respectively). Double-headed arrows indicate individual animals. Scale bars: 20 μm. (B) *cnx-1* reporter is induced in V5 seam-lineage–derived neuroblast cells in early L2 animals. Small arrows point to the seam cells outlines. Scale bar: 5 μm. (C) ER stress is able to induce expression of the *hsp-3* and *enpl-1* transcriptional reporters in seam cells and in hypodermis. The *hsp-3* reporter can be induced equally strongly in both anterior and posterior daughters of dividing seam cells in stressed animals. Small arrows point to seam-cell outlines. Animals were incubated on plates containing 10 μg/ml tunicamycin for 24 hours. DMSO (vehicle control)-treated animals were not different from untreated. Scale bars: 10 μm. AJM, Apical Junction Molecule; BiP, immunoglobulin heavy chain-binding protein; *daf-28(sa191)*, an allele causing ectopic L2d entry; GRP94, Glucose Regulated Protein, 94 kDa; ER, endoplasmic reticulum; ex, excretory cell; GFP, green fluorescent protein; HSP, Heat-Shock Protein; int, intestinal cell; L2, second larval stage; L2d, predauer L2; n, head neuron; ph, pharynx; UPR, unfolded protein response; vc, ventral cord neuron.
(TIF)

S4 Fig Regulatory elements in *hsp-4* promoter. (A) Schematic representation of the promoter used in p*hsp-4*::GFP reporter (gray line). Previously identified and putative regulatory elements/transcription factor binding sites are indicated relative to the coding region. Corresponding sequences, their positions, and orientation relative to the sense strain are indicated. (B) *hsp-4* reporter lacking either only the ERSE-II region (left panel) or both known ER stress elements (right panel) is still specifically induced in the differentiating alae-secreting cells. (C) Screenshot of the WormBase GBrowse image of BLMP-1 binding peak in *hsp-4* promoter, based on ModeEncode CHIP data. CHIP, Chromatin precipitation; ER, endoplasmic reticulum; GFP, green fluorescent protein; HSP-4, Heat-Shock Protein 4.
(TIF)

S5 Fig BLMP-1 represses both BiP isoforms but not other UPR targets. (A) Down-regulation of *blmp-1* results in mild induction of *hsp-3* expression in seam cells but not hypodermis of late L2d animals. RNAi and scoring as in Fig 3, the expression classes scored were induction in all seam cells (indicated as s.c.), induction in one or more but not in all seam cells (few s.c.), or no induction. (B) Down-regulation of *blmp-1* did not result in induction in seam cells of two additional UPR target genes, *enpl-1* and *cnx-1*, coding for *C*. *elegans* orthologues of GRP94 and calnexin, respectively. BiP, immunoglobulin heavy chain-binding protein; BLMP-1, a *C*. *elegans* orthologue of B-Lymphocyte-Induced Maturation Protein 1 BLIMP1; GRP94, Glucose Regulated Protein, 94 kDa; *hsp-3*, heat shock protein 3; L2, second larval stage; L2d, predauer L2; RNAi, RNA interference; UPR, unfolded protein response.
(TIF)

## Abbreviations

AJM-1: Apical Junction Molecule
ATF: Activating Transcription Factor
BiP: immunoglobulin heavy chain-Binding Protein
BLMP-1/BLIMP1: B-Lymphocyte-Induced Maturation Protein 1
bZIP: basic leucine-zipper
CGC: *Caenorhabditis* Genetics Center
CHO: Chinese hamster ovary
COL: collagen
CRE: c-AMP response element
CREB: CRE binding protein
DIC: differential interference contrast
ER: endoplasmic reticulum
FOXO3: Forkhead Box Protein O3
GFP: green fluorescent protein
*Grp78*: Glucose-Regulated Protein, 78 kDa
GRP94: Glucose Regulated Protein, 94 kDa
HIS: histone
HLH-1: helix–loop–helix 1
HSF-1: heat-shock transcription factor 1
HSP-4: Heat-Shock Protein 4
IGF: insulin-like growth factor
IRE1: Serine/Threonine-Protein Kinase/Endoribonuclease IRE1
IRF: interferon regulatory factor
L2: second larval
L2d: predauer L2
L3: third larval
L4: fourth larval
modENCODE: Model Organism Encyclopedia Of DNA Elements
MyoD: Myogenic Differentiation
NF-Y: Nuclear Transcription Factor Y
NIH: National Institutes of Health
OASIS: Old Astrocyte Specifically Induced Substance
PERK: PRKR-Like Endoplasmic Reticulum Kinase
PRD: positive regulatory domain
RNAi: RNA interference
STAT3: Signal Transducer and Activator Of Transcription 3
TGFβ: Transforming Growth Factor β
TLR: Toll-like receptor
UPR: unfolded protein response
VEGF: Vascular Endothelial Growth Factor
WT: wild type
XBP-1: X-Box Binding Protein 1
XBP-1s: spliced XBP-1
xHOT: extreme highly occupied target
Zf9: zinc finger 9
