# Supplementary figures and images for: HSP-4/BiP expression in secretory cells is regulated by a developmental program and not by the unfolded protein response
Source: PLoS Biol. 2019 Mar 25;17(3):e3000196. doi: 10.1371/journal.pbio.3000196 (PMC6448932; doi:10.1371/journal.pbio.3000196)

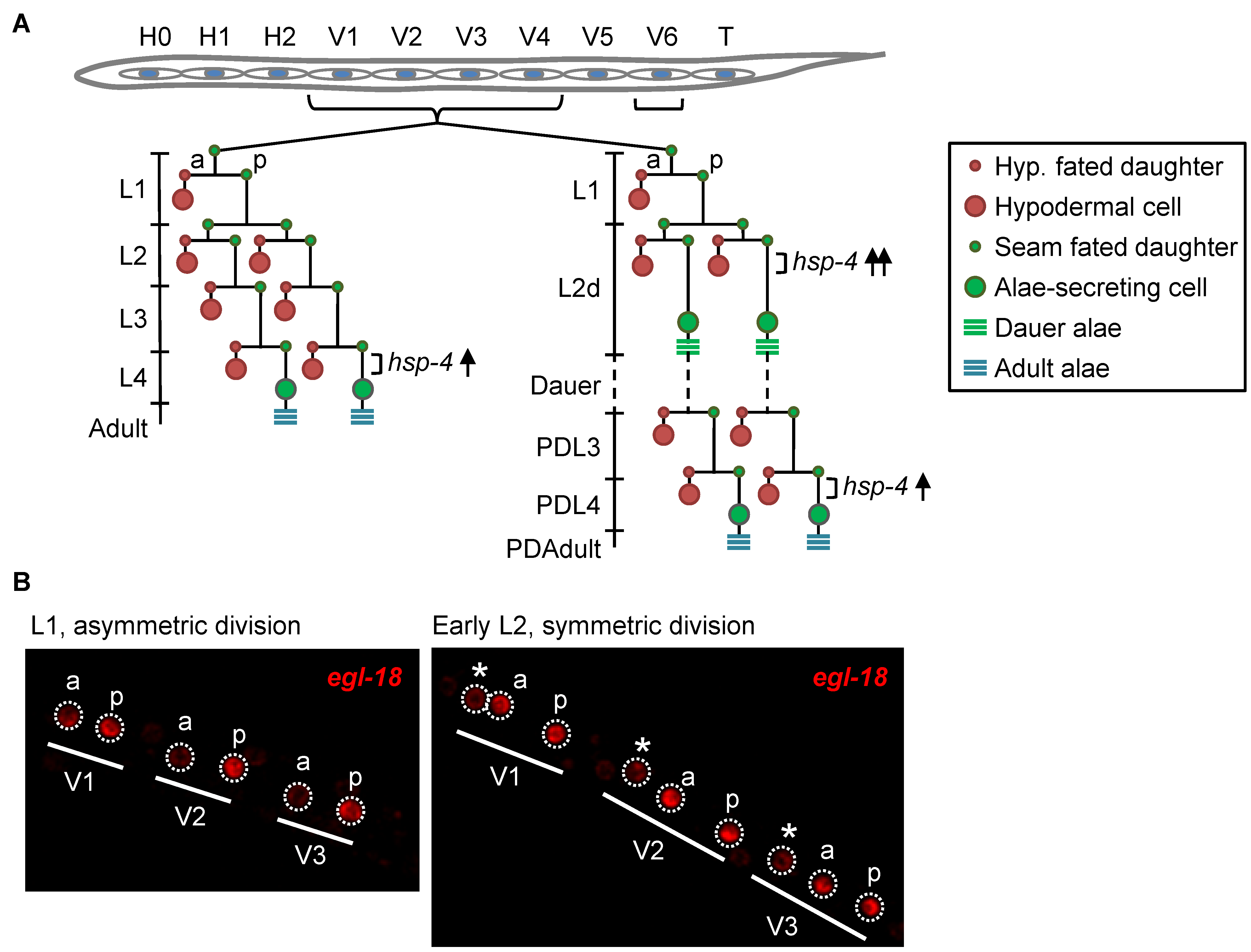

Supplement: S1 Fig — (A) Schematic diagram of cell divisions in V1–V4 and V6 seam lineages during postembryonic development. Positions and lineages of seam cells in L1 stage larvae are indicated in and above the worm outline. Left diagram corresponds to reproductive development, right to dauer development. After most asymmetric divisions, anterior daughter cells (a) fuse with the hyp7 hypodermal syncytial cell, while posterior (p) daughters retain their stem-like seam fate. After the last asymmetric division in the L4 stage, posterior daughters initiate terminal differentiation into the alae-secreting cell, fuse with each other, and begin to secrete alae-constituents and other cuticular proteins (indicated by blue horizontal lines). Additionally, posterior daughters may undergo differentiation into alae-secreting cells at the end of the L2d stage, resulting in secretion of the dauer cuticle (green horizontal lines); these cells do not fuse and resume asymmetric divisions in PD animals. (B) pegl-18::mCherry transgene in L1–L2 stage daf-28(sa191) animals. Left panel, L1 animal (20 hours postgastrula) shows differential expression of egl-18 reporter in seam cells following the first asymmetric division. Anterior (a) and posterior (p) daughters are indicated. Right panel, early L2 animal (26 hours postgastrula) following the symmetric division, with similar egl-18 expression in anterior and posterior daughters; stars indicate anterior daughters from the previous round of division in the L1 stage. daf-28(sa191), an allele causing ectopic L2d entry; pegl-18, egl-18 promoter, active in the posterior seam cells after asymmetric divisions; L1, first larval stage; L2, second larval stage; L2d, predauer L2; L4, fourth larval stage; PD, postdauer. (TIF) [file pbio.3000196.s002.tif]

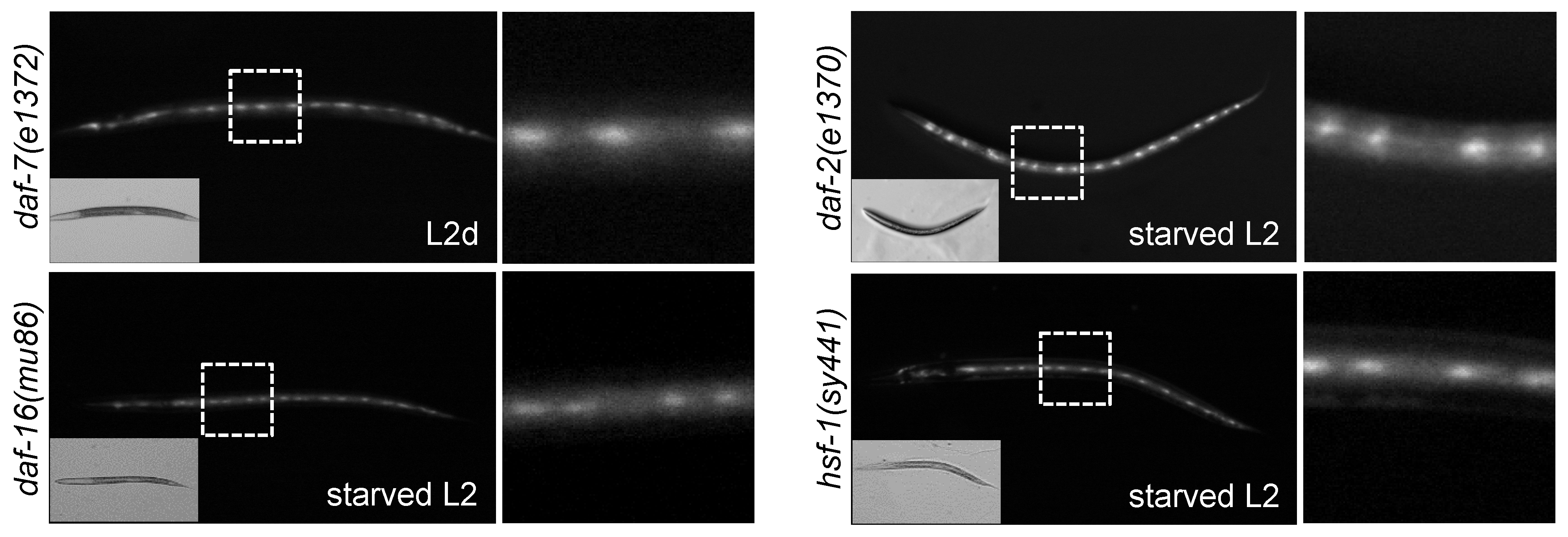

Supplement: S2 Fig — Fluorescence micrographs of predauer animals of indicated mutant strains, expressing phsp-4::GFP. daf-7(e1372) mutant animals enter the L2d stage at 20 °C, similar to the daf-28(sa191) animals; in other strains, the predauer stage was induced by starvation/crowding. Imaging as in Fig 1A; right panels are close views of the boxed areas. daf-28(sa191), an allele causing ectopic L2d entry; GFP, green fluorescent protein; HSP-4, Heat-Shock Protein 4; L2, second larval stage; L2d, predauer L2. (TIF) [file pbio.3000196.s003.tif]

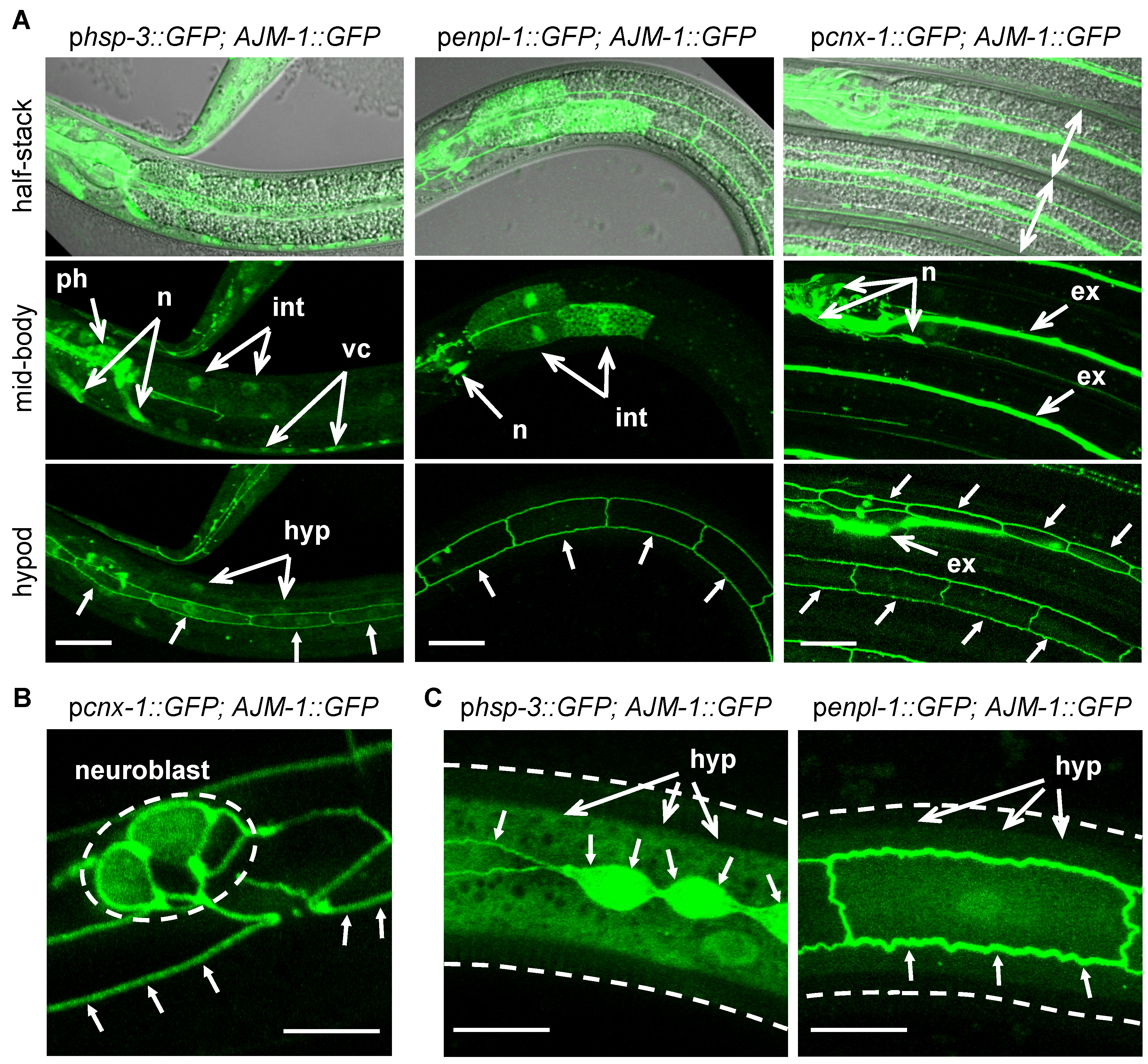

Supplement: S3 Fig — (A) Confocal micrographs of daf-28(sa191) L2d animals carrying indicated transgenes. Upper panels are projections of confocal stacks through half of the animal, overlaid on a transmitted light image; middle and bottom panels show projections of confocal stacks through the middle of the body or through the hypodermal layer. AJM-1::GFP protein marks apical junctions and outlines seam-cell boundaries (small closed arrows in the bottom panels). Open arrows point to various cells showing induction of the transcriptional reporters for indicated UPR-target genes (hsp-3, enpl-1, and cnx-1, coding for C. elegans orthologues of BiP, GRP94, and calnexin, respectively). Double-headed arrows indicate individual animals. Scale bars: 20 μm. (B) cnx-1 reporter is induced in V5 seam-lineage–derived neuroblast cells in early L2 animals. Small arrows point to the seam cells outlines. Scale bar: 5 μm. (C) ER stress is able to induce expression of the hsp-3 and enpl-1 transcriptional reporters in seam cells and in hypodermis. The hsp-3 reporter can be induced equally strongly in both anterior and posterior daughters of dividing seam cells in stressed animals. Small arrows point to seam-cell outlines. Animals were incubated on plates containing 10 μg/ml tunicamycin for 24 hours. DMSO (vehicle control)-treated animals were not different from untreated. Scale bars: 10 μm. AJM, Apical Junction Molecule; BiP, immunoglobulin heavy chain-binding protein; daf-28(sa191), an allele causing ectopic L2d entry; GRP94, Glucose Regulated Protein, 94 kDa; ER, endoplasmic reticulum; ex, excretory cell; GFP, green fluorescent protein; HSP, Heat-Shock Protein; int, intestinal cell; L2, second larval stage; L2d, predauer L2; n, head neuron; ph, pharynx; UPR, unfolded protein response; vc, ventral cord neuron. (TIF) [file pbio.3000196.s004.tif]

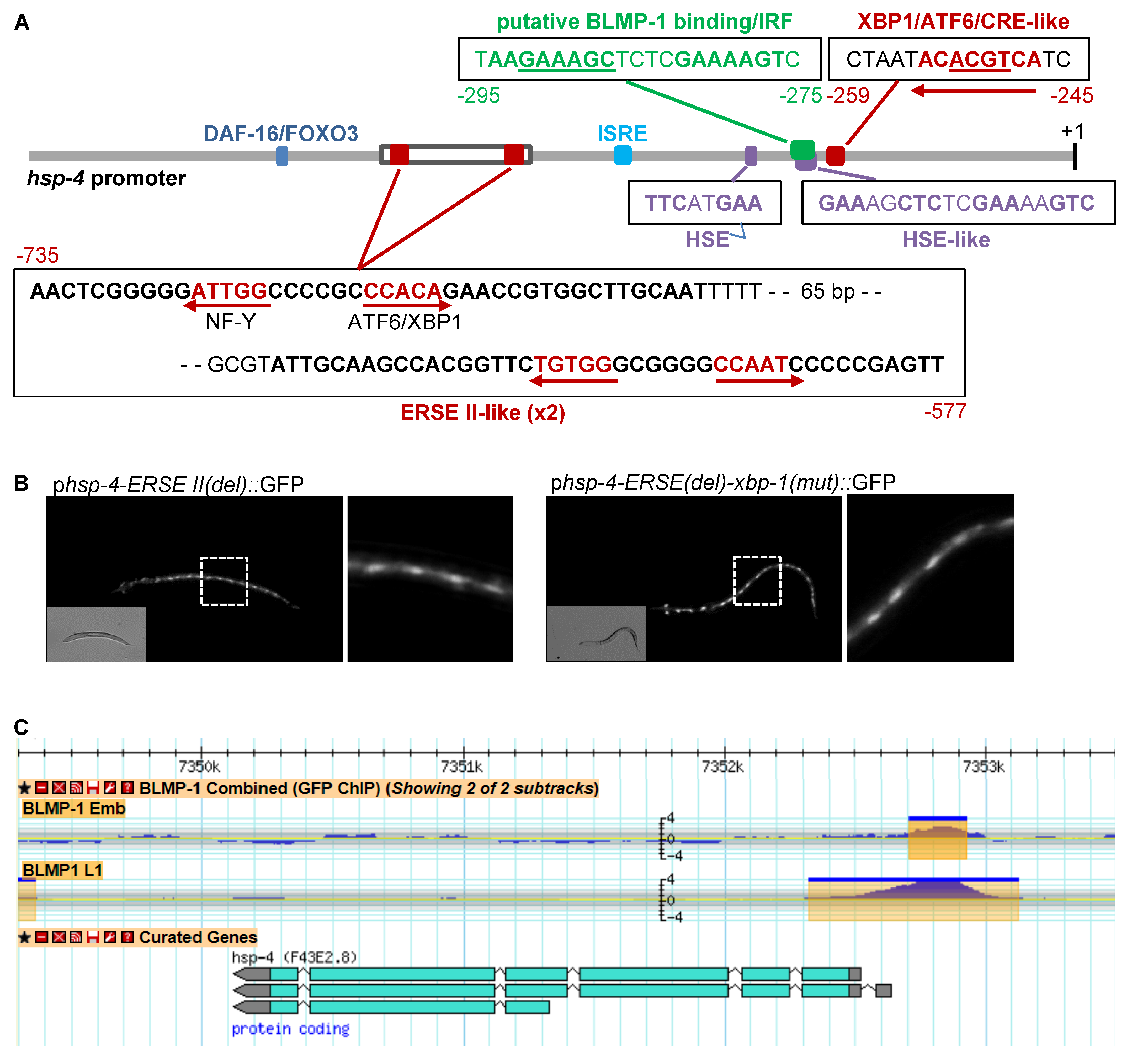

Supplement: S4 Fig — (A) Schematic representation of the promoter used in phsp-4::GFP reporter (gray line). Previously identified and putative regulatory elements/transcription factor binding sites are indicated relative to the coding region. Corresponding sequences, their positions, and orientation relative to the sense strain are indicated. (B) hsp-4 reporter lacking either only the ERSE-II region (left panel) or both known ER stress elements (right panel) is still specifically induced in the differentiating alae-secreting cells. (C) Screenshot of the WormBase GBrowse image of BLMP-1 binding peak in hsp-4 promoter, based on ModeEncode CHIP data. CHIP, Chromatin precipitation; ER, endoplasmic reticulum; GFP, green fluorescent protein; HSP-4, Heat-Shock Protein 4. (TIF) [file pbio.3000196.s005.tif]

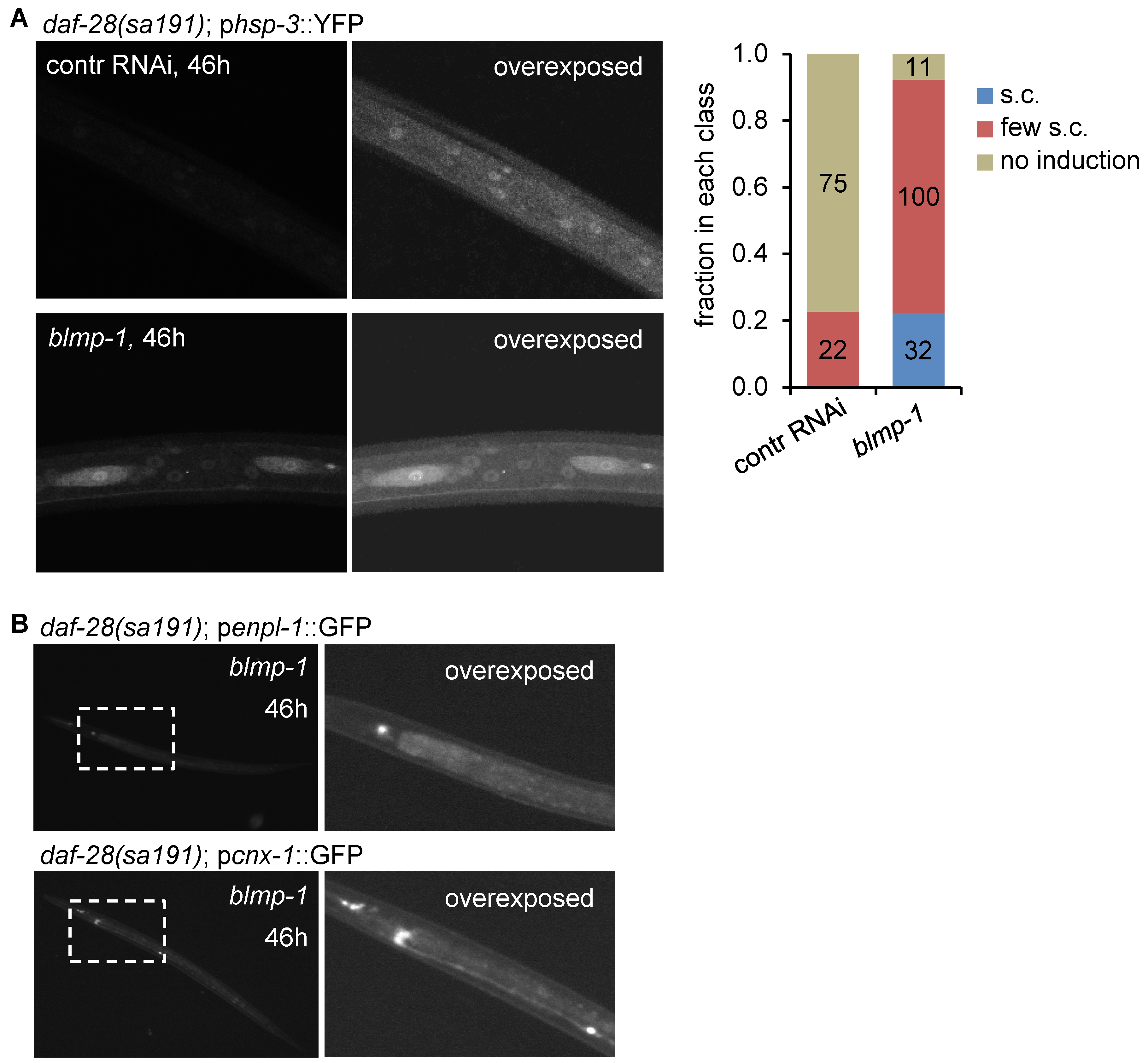

Supplement: S5 Fig — (A) Down-regulation of blmp-1 results in mild induction of hsp-3 expression in seam cells but not hypodermis of late L2d animals. RNAi and scoring as in Fig 3, the expression classes scored were induction in all seam cells (indicated as s.c.), induction in one or more but not in all seam cells (few s.c.), or no induction. (B) Down-regulation of blmp-1 did not result in induction in seam cells of two additional UPR target genes, enpl-1 and cnx-1, coding for C. elegans orthologues of GRP94 and calnexin, respectively. BiP, immunoglobulin heavy chain-binding protein; BLMP-1, a C. elegans orthologue of B-Lymphocyte-Induced Maturation Protein 1 BLIMP1; GRP94, Glucose Regulated Protein, 94 kDa; hsp-3, heat shock protein 3; L2, second larval stage; L2d, predauer L2; RNAi, RNA interference; UPR, unfolded protein response. (TIF) [file pbio.3000196.s006.tif]
